# William D. Bizzell, M. D.

**Published:** 1890-07

**Authors:** 


### DR. WILLIAM D. BIZZELL.

As we go to press we learn with sorrow of the death of Dr. W. D. Bizzell, of Atlanta, which occurred on the 30th of June. Dr. Bizzell had been in feeble health for several months and his death was not unexpected. For about ten years he practiced his profession in this city, and his ability as a physician commanded the respect of the profession and the public, while his genial disposition and gentlemanly manners endeared him to the hearts of a large circle of patients and friends. He was a fluent writer, and made frequent and valuable contributions to medical literature. As a lecturer he displayed marked ability, and and filled with success for several years the Chair of Principles Practice of Medicine in the Southern Medical College. He leaves a wife and two daughters to whom the JOURNAL extends its heartfelt sympathy.

---

A FULL report of resolutions passed by the profession upon the deaths] of Drs. Westmoreland and Bizzell will appear in next issue of THE JOURNAL.

